# The effects of wrist motion and hand orientation on muscle forces: A physiologic wrist simulator study

**DOI:** 10.1016/j.jbiomech.2017.06.017

**Published:** 2017-07-26

**Authors:** Darshan S. Shah, Claire Middleton, Sabahat Gurdezi, Maxim D. Horwitz, Angela E. Kedgley

**Affiliations:** a Department of Bioengineering, Imperial College London, London, United Kingdom; b Department of Hand Surgery, Chelsea and Westminster Hospital, London, United Kingdom

**Keywords:** Simulator, Control, Muscle forces, Hand orientation, Circumduction

## Abstract

Although the orientations of the hand and forearm vary for different wrist rehabilitation protocols, their effect on muscle forces has not been quantified. Physiologic simulators enable a biomechanical evaluation of the joint by recreating functional motions in cadaveric specimens. Control strategies used to actuate joints in physiologic simulators usually employ position or force feedback alone to achieve optimum load distribution across the muscles. After successful tests on a phantom limb, unique combinations of position and force feedback – hybrid control and cascade control – were used to simulate multiple cyclic wrist motions of flexion-extension, radioulnar deviation, dart thrower’s motion, and circumduction using six muscles in ten cadaveric specimens. Low kinematic errors and coefficients of variation of muscle forces were observed for planar and complex wrist motions using both novel control strategies. The effect of gravity was most pronounced when the hand was in the horizontal orientation, resulting in higher extensor forces (*p* < 0.017) and higher out-of-plane kinematic errors (*p* < 0.007), as compared to the vertically upward or downward orientations. Muscle forces were also affected by the direction of rotation during circumduction. The peak force of flexor carpi radialis was higher in clockwise circumduction (*p* = 0.017), while that of flexor carpi ulnaris was higher in anticlockwise circumduction (*p* = 0.013). Thus, the physiologic wrist simulator accurately replicated cyclic planar and complex motions in cadaveric specimens. Moreover, the dependence of muscle forces on the hand orientation and the direction of circumduction could be vital in the specification of such parameters during wrist rehabilitation.

## 1 Introduction

Physiologic simulators recreate the kinematic and kinetic conditions of the natural wrist joint in vitro by applying tensile loads to tendons of cadaveric specimens (Erhart et al., 2012, Werner et al., 1996). This enables the identification of key variables affecting biomechanics, with direct implications for surgical reconstructions and/or rehabilitation procedures. In the case of wrist rehabilitation, one proposed standard protocol includes placing the forearm in the horizontal orientation with neutral pronation (Fess and Moran, 1981), followed by the performance of prescribed exercises (Williams et al., 2001). However, some rehabilitation protocols are comprised of a larger set of wrist motions with varying forearm rotation and hand orientations (Pandian and Narayan, 2012). Therefore, it is important to quantify the effects of forearm rotation and hand orientations on wrist biomechanics, especially since they are known to affect key factors like grip strength, while assessing the progress of wrist rehabilitation (Richards et al., 1996). Although the effects of varying the forearm pronation-supination angle on wrist muscle forces have been studied in vitro (Farr et al., 2013), all reported wrist simulators in the literature have quantified wrist biomechanics only in the vertical orientation of the hand (Erhart et al., 2012, Werner et al., 1996); the effects of varying hand orientations on wrist muscle forces have not been explored. Moreover, while simulating complex functional motions of the wrist, only one of the directions of circumduction has been analysed on a wrist simulator (Werner et al., 1996, Werner et al., 2010); the effect of changing the direction of circumduction on wrist muscle forces has not been analysed.

In order to simulate planar and complex wrist motions in vitro using a physiologic simulator, the resolution of the load distribution between the tendons is vital, owing to an indeterminate problem of redundant muscle actuation, which arises due to the presence of six primary muscles controlling two degrees of rotation in the wrist. Some physiologic joint simulators recreate joint motion by controlling the excursion of one of the muscles, designated as the ‘prime mover’, while the remaining muscles are controlled using prescribed forces calculated as a proportion of the prime mover force, based on combinations of muscle architecture parameters and/or electromyographic (EMG) signals (Johnson et al., 2000, Kedgley et al., 2007, Nishiwaki et al., 2014, Sharkey and Hamel, 1998). Physiologic wrist simulators have either employed position feedback to control agonists and force feedback to control antagonists (Werner et al., 1996), or predefined sets of force profiles corresponding to specific wrist motions to control the wrist muscles (Erhart et al., 2012). Cascade control, which has been demonstrated computationally but not implemented experimentally, uses a combination of position and force feedback as an alternative method of controlling the joint motion (Colbaugh and Glass, 1993). In previous work, hybrid control and cascade control – two novel control strategies combining position and force feedback – were implemented on a functional replica of the human forearm and hand, and resulted in more accurate kinematics and more physiologic muscle forces as compared to the conventional position and force control strategies (Shah and Kedgley, 2016).

Therefore, the first objective of this study was to test the robustness and repeatability of these novel control strategies in recreating simple and complex wrist motions in cadaveric specimens. The second objective of this study was to quantify the effects of varying hand orientations on wrist biomechanics, with the hypothesis that wrist muscle forces would be higher for the horizontal orientation. The third objective of this study was to quantify the muscle forces during wrist circumduction, with the hypothesis that the direction of motion would significantly affect the magnitude of the forces.

## 2 Materials and methods

### 2.1 Specimens

Ten fresh-frozen cadaveric specimens (eight females and two males, aged 49.7 ± 10.4 years) were obtained from a licensed human tissue facility. Ethical approval for the use of these specimens was obtained from the Tissue Management Committee of the Imperial College Healthcare Tissue Bank, according to the Human Tissue Act. Patients had no history of relevant wrist disorders. The specimens, stored at −20 °C prior to this study, were thawed at room temperature for 12 h. All soft tissue was resected 5 cm proximal to the wrist, except for the six wrist muscles considered for this study – flexor carpi radialis (FCR), flexor carpi ulnaris (FCU), extensor carpi radialis longus (ECRL), extensor carpi radialis brevis (ECRB), extensor carpi ulnaris (ECU), and abductor pollicis longus (APL) – which were dissected at their distal musculotendinous junction. Care was taken to preserve the ligamentous structures at the elbow and the forearm interosseous membrane. Kirschner-wires were used to fix the elbow in 90° flexion and the forearm in 0° pronation. A stainless-steel stud in the intramedullary canal of the humerus allowed fixation of the specimen to the simulator.

### 2.2 Simulator design

**Fig. 1:**
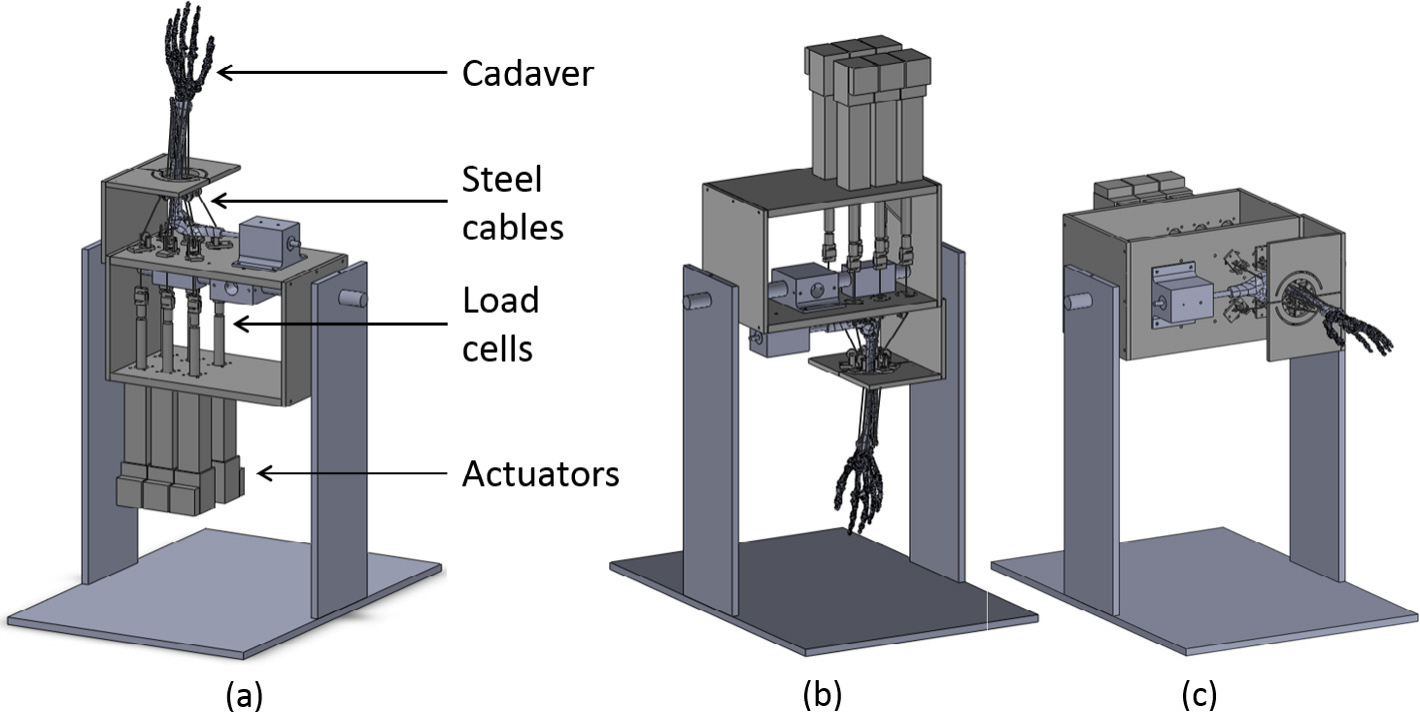
A schematic diagram of the wrist simulator with the hand in different orientations – (a) vertically upward (b) vertically downward (c) horizontal.

Motion at the wrist was recreated by applying tensile loads using linear actuators (SMS Machine Automation, Barnsley, UK) mounted in-line with servo motors (Animatics Corp., Milpitas, USA) via steel cables guided through custom pulleys and sutured to distal tendons of aforementioned muscles (Fig. 1a). Load cells (Applied Measurements Ltd., UK) were connected in series with the actuators to monitor force applied to each tendon. Clusters of retroreflective passive markers were fixed rigidly to the third metacarpal and the radius to define the co-ordinate systems of the hand and the forearm, respectively, using anatomical landmarks recommended by the ISB (Wu et al., 2005). An eight-camera optical motion capture system (Qualisys, Göteborg, Sweden) was used to obtain the joint angles in real time.

### 2.3 Simulations

Each wrist was manually moved over the entire passive range of motion of flexion-extension (FE) and radioulnar deviation (RUD), with actuators applying a constant load of 10 N to each tendon to prevent them from unloading. The joint kinematics and corresponding actuator displacements were used to determine the muscle moment arms using the tendon excursion method (An et al., 1983). The mean moment arms over the range of motion were used as specimen-specific inputs to hybrid and cascade control, which were implemented using custom-written LabVIEW (National Instruments, Austin, USA) codes to simulate active motions of the wrist. Hybrid control utilised position feedback to drive joint kinematics, and simultaneous force feedback to ensure muscle forces remain within physiologic bounds. The lower bound on muscle forces was chosen according to the minimum muscle activity obtained from EMG (Fagarasanu et al., 2004), while the upper bound was defined as the product of muscle physiological cross-sectional area (Holzbaur et al., 2007) and specific tension (Kent-Braun and Ng, 1999). Cascade control included force control nested within position control, with a quadratic optimisation routine to calculate muscle forces required to obtain the desired kinematics. The objective of the optimisation routine in cascade control was to minimise the sum of the square of the muscle stresses, with one of the constraints being on the muscle impedance (*ρ*), defined as the sum of all muscle forces (Colbaugh and Glass, 1993). *ρ* was allowed to vary between 0 N and a relatively large value of 400 N, in order to leave the sum of the muscle forces in cascade control effectively unconstrained. All other input parameters in hybrid and cascade control were maintained from the previous study (Shah and Kedgley, 2016).

Multiple cyclic planar motions – FE of amplitude 30° (FE-30) and RUD of amplitude 10° (RUD-10) – were simulated using hybrid control and cascade control to compare the kinematic errors and repeatability across the two control strategies. To assess the effects of gravity on the wrist muscle forces, planar motions of FE-30 and RUD-10 were tested in hybrid control with the hand in three orientations – vertically upward (hand above the elbow), vertically downward (hand below the elbow) and horizontal (palm facing down) (Fig. 1).

Complex cyclic motions – dart thrower’s motion (DTM) (20° extension with 15° radial deviation to 20° flexion with 15° ulnar deviation), clockwise circumduction (CCD_cw_) (30° flexion to 10° ulnar deviation to 30° extension to 10° radial deviation) and anticlockwise circumduction (CCD_acw_) (30° flexion to 10° radial deviation to 30° extension to 10° ulnar deviation) – were simulated using hybrid and cascade control in the vertically upward orientation.

### 2.4 Data analysis

Each specimen was moved through five cycles for all wrist motions. The kinematic accuracy, defined as the mean of the absolute error between the actual and the desired joint angles across the range of motion, was used as an indicator of the robustness of the control strategy. Muscle forces were evaluated as a function of joint kinematics, at every 10° in FE and 5° in RUD. The standard deviations of muscle forces for the five cycles of each wrist motion were computed as a function of kinematics, and the mean of these across 10 specimens was used as a measure of repeatability.

Non-parametric tests were used to compare the data since they were found to deviate from a normal distribution when checked for normality using the Shapiro-Wilk test (IBM SPSS Statistics, IBM Corp., Armonk, USA). The Wilcoxon-signed rank test was used to compare data between two groups, while the Friedman test was used to compare data across three groups (significance: *p* < 0.05). If significant interactions were observed in the Friedman test, a Wilcoxon-signed rank test was performed, with a Bonferroni adjustment for multiple comparisons, to see pairwise differences (significance: *p* < 0.017).

## 3 Results

**Fig. 2:**
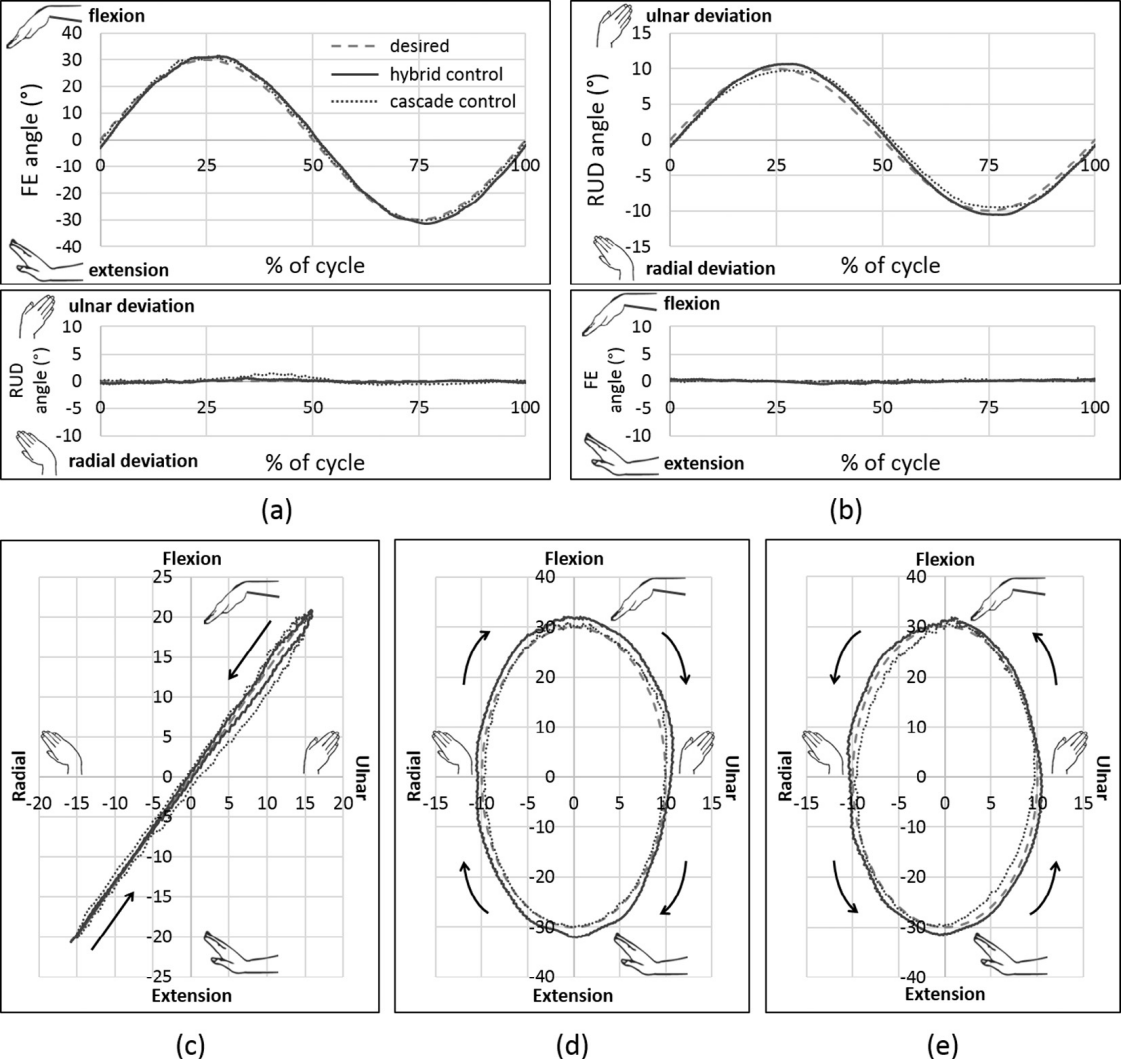
In-plane and out-of-plane kinematics for planar motions – (a) flexion-extension and (b) radioulnar deviation – and trajectories of complex motions – (c) dart thrower’s motion (d) clockwise circumduction and (e) anticlockwise circumduction – in hybrid control (solid lines) and cascade control (dotted lines), as compared to the desired kinematics and trajectories (dashed lines), with the hand in the vertically upward orientation, for a representative cycle of one of the specimens (*X*-axis represents radioulnar deviation, *Y*-axis represents flexion-extension).

Both hybrid and cascade control resulted in accurate joint kinematics (Fig. 2), with low in-plane and out-of-plane kinematic errors for the planar motions FE-30 and RUD-10, as well as low kinematic errors for complex motions DTM, CCD_cw_ and CCD_acw_ (Appendix A.1). Repeatable muscle forces were obtained using both hybrid and cascade control for all six muscles (Appendix A.2).

**Fig. 3:**
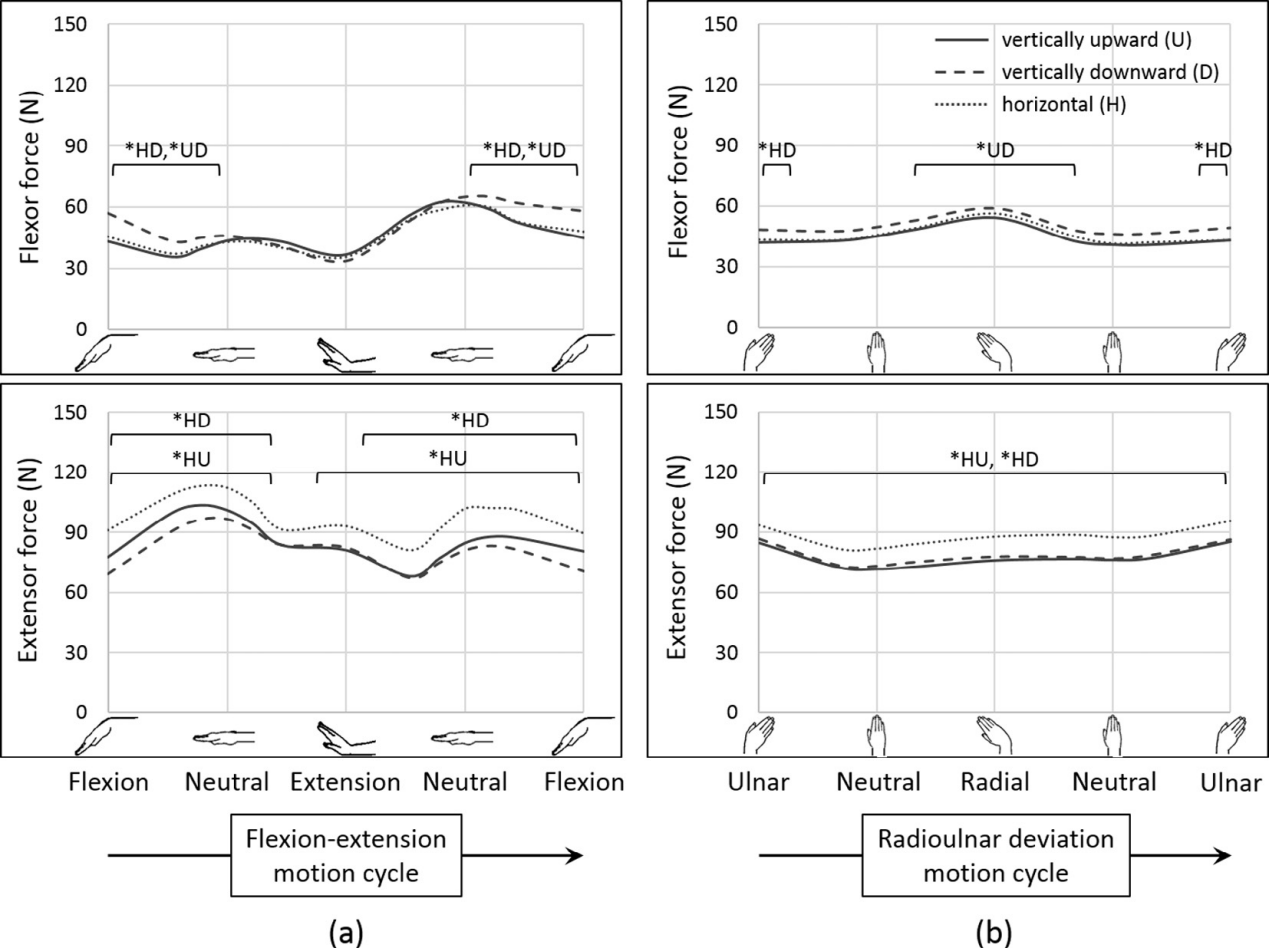
Flexor force (sum of flexor carpi radialis, flexor carpi ulnaris, and abductor pollicis longus forces) and extensor force (sum of extensor carpi radialis longus, extensor carpi radialis brevis, and extensor carpi ulnaris forces) for (a) flexion-extension of 30° (FE-30) and (b) radioulnar deviation of 10° (RUD-10) in hybrid control with the hand in the vertically upward (U), vertically downward (D) and horizontal (H) orientations. The asterisk (*) represents statistically significant pairwise differences between pairs of orientations of the hand (*p* < 0.017).

No differences were observed between kinematic errors in wrist motions simulated with the hand in the vertically upward and downward orientations (*p*-value of the vertically upward orientation compared to the vertically downward orientation (*p*_UD_) > 0.169); however, the horizontal orientation resulted in higher out-of-plane errors in both FE-30 and RUD-10 compared to the vertically upward and downward orientations (*p*-value of the horizontal orientation compared to the vertically upward orientation (*p*_HU_) < 0.007, *p*-value of the horizontal orientation compared to the vertically downward orientation (*p*_HD_) < 0.005) (Table 1). The extensor force (sum of ECRL, ECRB, ECU) was higher for the hand in the horizontal orientation throughout the range of motion in FE and RUD (*p*_HU_ < 0.017, *p*_HD_ < 0.017), except at small angles during extension (*p*_HU_ > 0.028, *p*_HD_ > 0.059) (Fig. 3). No differences were found in the extensor force between the vertically upward and downward orientations in FE and RUD (*p*_UD_ > 0.022). The flexor force (sum of FCR, FCU, APL) was higher for the hand in the vertically downward orientation than for either the vertically upward or horizontal orientations during flexion greater than 10° (*p*_UD_ < 0.009, *p*_HD_ < 0.005). The flexor force was also higher for the hand in the vertically downward orientation than in the vertically upward orientation for radial deviation greater than 5° (*p*_UD_ < 0.017) and than in the horizontal orientation for ulnar deviation of 10° (*p*_HD_ < 0.013). No differences were found in the flexor force between the horizontal and vertically upward orientations in either FE or RUD (*p*_HU_ > 0.074).

**Table 1:** Mean kinematic errors during flexion-extension of ±30° (FE-30) and radioulnar deviation of ±10° (RUD-10) in hybrid control with the hand in the vertically upward (U), vertically downward (D) and horizontal (H) orientations. Data are represented as mean ± one standard deviation across 10 specimens. Standard deviations of less than 0.05° have been reported as 0.0°. (*p*_UD_ = *p*-value of the vertically upward orientation compared to the vertically downward orientation, *p*_HU_ = *p*-value of the horizontal orientation compared to the vertically upward orientation, *p*_HD_ = *p*-value of the horizontal orientation compared to the vertically downward orientation; significance: *p* < 0.017).

Motion	Hand orientation	Mean error in FE (°)	Mean error in RUD (°)
FE-30	Vertically upward	1.8 ± 0.3	0.2 ± 0.0
	Vertically downward	1.7 ± 0.3	0.2 ± 0.0
	*p*_UD_	0.508	0.333
	Horizontal	2.0 ± 0.4	0.8 ± 0.6
	*p*_HU_	0.139	0.005
	*p*_HD_	0.047	0.005

RUD-10	Vertically upward	0.2 ± 0.1	0.6 ± 0.1
	Vertically downward	0.2 ± 0.1	0.6 ± 0.1
	*p*_UD_	0.508	0.169
	Horizontal	0.6 ± 0.4	0.8 ± 0.4
	*p*_HU_	0.007	0.017
	*p*_HD_	0.005	0.013

**Fig. 4:**
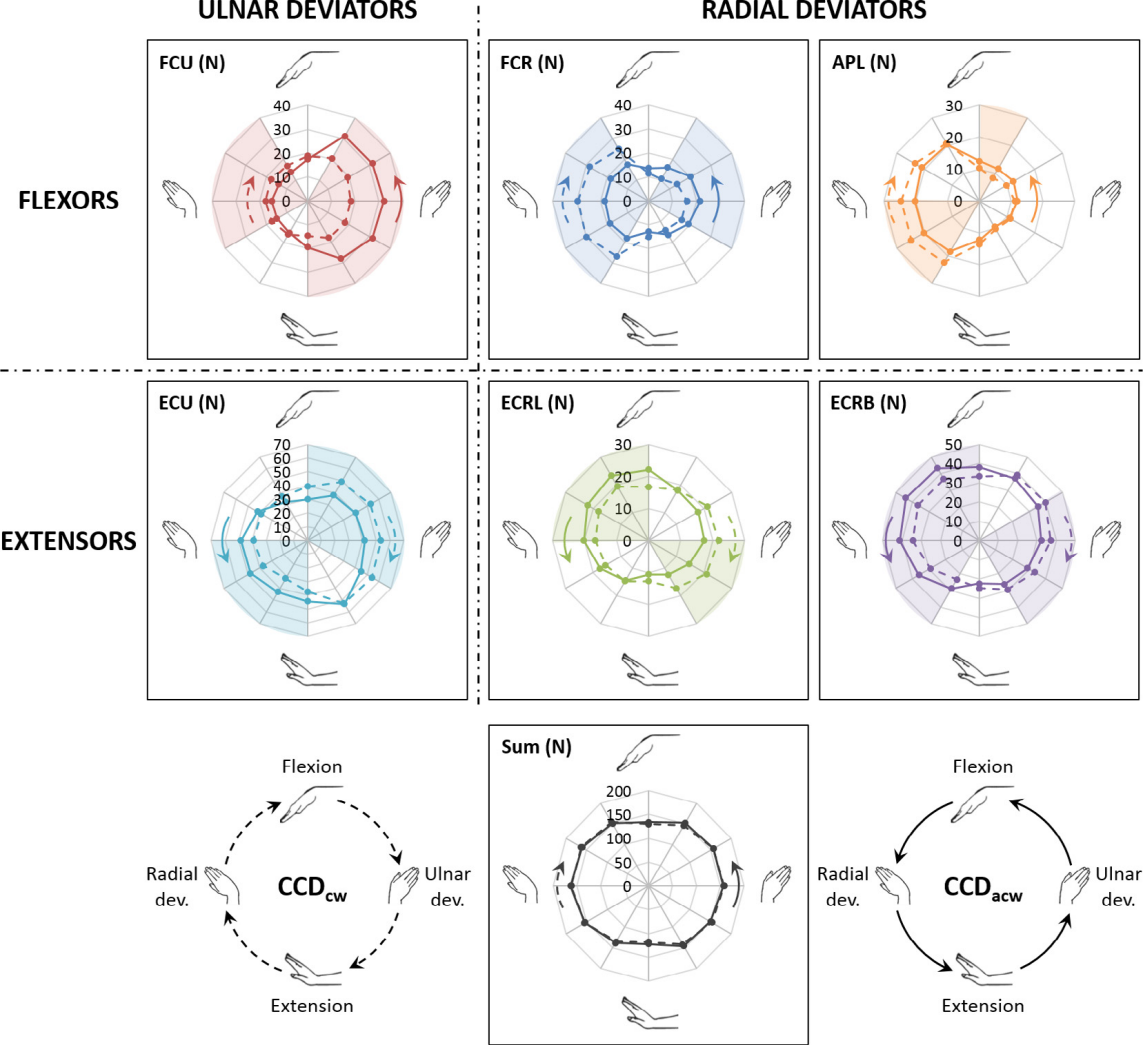
Muscle forces (N) of the flexor carpi ulnaris (FCU), flexor carpi radialis (FCR), abductor pollicis longus (APL), extensor carpi ulnaris (ECU), extensor carpi radialis longus (ECRL), extensor carpi radialis brevis (ECRB), and the sum of all muscle forces for clockwise circumduction (CCD_cw_) (dashed lines) and anticlockwise circumduction (CCD_acw_) (solid lines) with the hand in vertically upward orientation in hybrid control. The shaded region represents statistically significant differences between the two directions of circumduction (*p* < 0.05).

The force profiles for FCR and FCU differed for CCD_cw_ and CCD_acw_ (Fig. 4) with the peak force and the mean force of the FCR higher in CCD_cw_ by 27% (*p* = 0.017) and 11% (*p* = 0.047), respectively, and the peak force and the mean force of the FCU higher in CCD_acw_ by 40% (*p* = 0.013) and 20% (*p* = 0.017), respectively. Although the force profiles for ECRL, ECRB, ECU and APL also differed for CCD_cw_ and CCD_acw_, no statistical difference was observed between their peak forces and mean forces (*p* > 0.074). No statistical difference was observed between the sum of all muscle forces throughout CCD_cw_ and CCD_acw_ (*p* > 0.203).

## 4 Discussion

A physiologic wrist simulator was developed to replicate motions of the intact human wrist in cadaveric specimens. One of the main advantages of employing an optical motion capture system to track joint angles was the use of light, passive markers, which did not appreciably add to the mass of the body segments. Hybrid and cascade control, which combined both position and force feedback simultaneously, resulted in accurate (Appendix A.1) and repeatable (Appendix A.2) wrist motions in vitro, thus supporting our hypothesis. However, owing to higher cycle times (Appendix A.1), lower repeatability (Appendix A.2) and large muscle forces (Appendix A.3) in cascade control, hybrid control was preferred over cascade control. One of the limitations of the simulator was the exclusion of the extrinsic muscles of the fingers and the thumb. Although these muscles pass through the wrist, and are therefore expected to affect wrist biomechanics, their inclusion would have further added to the complexity of the control strategies. Another limitation of the study was the fixation of the ulna relative to the radius, which prevented forearm pronation-supination or ulnar length variation during wrist motions.

Differences in muscle forces observed for the hand in different orientations indicated that the gravity vector affected the muscle forces. The external moment due to gravity was maximal when the hand was in the horizontal orientation with a neutral wrist position (FE = 0°, RUD = 0°) because the gravity vector acted perpendicular to the long axis of the hand in this orientation. Moreover, with the palm faced down in this orientation, the extensors had to generate more force to counteract gravity. Hence, in FE, the extensor force in this orientation was higher than in the vertically upward or downward orientations (Fig. 3a). Even in the case of RUD, the extensor forces were higher in the horizontal orientation, since they acted to maintain FE = 0° as well as perform the desired RUD, which meant counteracting gravity throughout the range of motion (Fig. 3b). Thus, when the hand is held in the horizontal orientation, depending on which direction gravity is acting (palmar, dorsal, radial or ulnar), the corresponding functional muscle group (extensors, flexors, ulnar deviators or radial deviators) will likely be overloaded, consistent with our hypothesis. Furthermore, the out-of-plane kinematic errors were higher for the hand in the horizontal orientation than the two vertical orientations (Table 1), suggesting that it is more difficult to control the wrist in the secondary degree of freedom as the influence of an external force increases, as is also reported in the control of robotic manipulators (Raibert and Craig, 1981). When comparing the two vertical orientations, the flexor force was higher in the vertically downward orientation for higher flexion angles (*p*_UD_ < 0.009) (Fig. 3a) to counteract gravity acting dorsally, while the extensor force was higher in the vertically upward orientation for higher flexion angles to counteract gravity acting palmarly, although the differences were not significant (*p*_UD_ > 0.017). These observations could support the placement of the forearm in the vertically upward orientation, as opposed to the proposed horizontal orientation (Fess and Moran, 1981), during the implementation of wrist rehabilitation protocols.

Although a difference in forces was observed for all muscles between CCD_cw_ and CCD_acw_ for some parts of the range of motion (Fig. 4), the sum of all muscle forces did not change with the direction of circumduction (*p* > 0.203). However, the peak and mean forces differed between CCD_cw_ and CCD_acw_ only for the FCR and FCU. Since FCR is a radial flexor of the wrist, the FCR force was higher in CCD_cw_ when the hand moved from extension to flexion via radial deviation. In contrast, since FCU is an ulnar flexor of the wrist, the FCU force was higher in CCD_acw_ when the hand moved from extension to flexion via ulnar deviation. Thus, muscle forces were dependent on the direction of circumduction, which could support the implementation or prohibition of one of these motions in rehabilitation protocols to overcome pathologies affecting either one of the FCR or the FCU, like tendinitis.

In conclusion, a physiologic wrist simulator was developed to accurately replicate cyclic planar and complex motions in cadaveric specimens using robust and repeatable control strategies. Factors such as the orientation of the hand, as well as the direction of complex motions, affected the wrist muscle forces, which could influence wrist rehabilitation protocols.

## Conflict of interest statement

The authors have no conflicts of interest to declare.
