# Supplementary material for: The effects of wrist motion and hand orientation on muscle forces: A physiologic wrist simulator study
Source: J Biomech. 2017 Jul 26;60:232–7. doi: 10.1016/j.jbiomech.2017.06.017 (PMC5555257; doi:10.1016/j.jbiomech.2017.06.017)
Supplement: Supplementary data 1 [file mmc1.docx]

**The effects of wrist motion and hand orientation on muscle forces: a physiologic wrist simulator study**

Darshan S Shah, Claire Middleton, Sabahat Gurdezi, Maxim D Horwitz, Angela E Kedgley

## Appendix A: Comparing hybrid control and cascade control

### A.1 – Cycle time and kinematic error

Hybrid control and cascade control were used to simulate multiple cyclic planar motions – flexion-extension of amplitude 30° (FE-30) and radioulnar deviation of amplitude 10° (RUD-10) in cadaveric wrists (Fig. 2). They were also used to simulate complex motions, such as dart thrower’s motion (DTM), clockwise circumduction (CCD_cw_) and anticlockwise circumduction (CCD_acw_). The cycle time for all wrist motions in hybrid control was lower than that in cascade control (Table A1).

In the case of both hybrid and cascade control, in-plane errors were less than 2°, and out-of-plane errors were less than 0.5° for planar motions, while the errors in both FE and RUD were less than 2° for complex motions. Low kinematic errors for both planar and complex motions established the accuracy, and hence the robustness, of hybrid and cascade control.

Hybrid control resulted in lower mean errors in RUD for planar motions (p<0.037), while cascade control resulted in lower mean errors in FE for all wrist motions (p<0.037) (Table A1). However, one of the drawbacks of cascade control was the higher cycle time as compared to hybrid control. Despite the high cycle time, cascade control resulted in high muscle forces (Appendix A.3), probably owing to the optimisation routine it employed. An attempt at reducing the cycle time or increasing the active range of motion resulted in an increase in the torque input to the optimisation routine, due to the increase in kinematic error, which led to a further rise in muscle forces. Hence, hybrid control was preferred over cascade control to simulate active wrist motions in vitro.

Table A1: Cycle times and mean kinematic errors during cyclic motions in hybrid and cascade control with the hand in the vertically upward orientation. Data are represented as mean ± one standard deviation across ten specimens. Standard deviations of less than 0.05° have been reported as 0.0°. p-values show differences between hybrid and cascade control (significance: p<0.05)

| **Motion** | **Control strategy** | **Cycle time (s)** | **mean error in FE (°)** | **mean error in RUD (°)** |
| --- | --- | --- | --- | --- |
| Flexion-extension  (FE)  ±30° | Hybrid | 10 | 1.8 ± 0.3 | 0.2 ± 0.0 |
|  | Cascade | 20 | 0.7 ± 0.2 | 0.4 ± 0.2 |
|  | p-value | - | 0.005 | 0.007 |
| Radioulnar deviation  (RUD)  ±10° | Hybrid | 10 | 0.2 ± 0.1 | 0.6 ± 0.1 |
|  | Cascade | 20 | 0.3 ± 0.2 | 0.8 ± 0.1 |
|  | p-value | - | 0.037 | 0.037 |
| Dart thrower’s motion  (DTM)  ±20° FE with ±15° RUD | Hybrid | 10 | 1.0 ± 0.2 | 0.8 ± 0.2 |
|  | Cascade | 20 | 0.4 ± 0.1 | 0.8 ± 0.1 |
|  | p-value | - | 0.008 | 0.594 |
| Clockwise circumduction  (CCD_cw_)  ±30° FE with ±10° RUD | Hybrid | 10 | 1.8 ± 0.3 | 0.6 ± 0.1 |
|  | Cascade | 35 | 0.5 ± 0.1 | 0.4 ± 0.2 |
|  | p-value | - | 0.012 | 0.123 |
| Anticlockwise circumduction  (CCD_acw_)  ±30° FE with ±10° RUD | Hybrid | 10 | 1.7 ± 0.2 | 0.7 ± 0.1 |
|  | Cascade | 35 | 0.5 ± 0.1 | 0.6 ± 0.2 |
|  | p-value | - | 0.012 | 0.263 |

### A.2 – Repeatability of muscle forces

Each intact specimen was moved through five cycles for every planar and complex wrist motion. Muscle forces were evaluated as a function of joint kinematics, at every 10° in FE and 5° in RUD. The standard deviations of muscle forces for the five cycles of each wrist motion were computed as a function of kinematics, and the mean of these across 10 specimens was used as a measure of repeatability. The coefficient of variation, defined as the ratio of the repeatability to the mean force across 10 specimens, was calculated as a function of joint kinematics and then averaged out across the complete range of motion (Table A2).

Muscle forces obtained using hybrid control were more repeatable than those obtained from cascade control for all six muscles for flexion angles greater than 10° in FE-30, DTM and CCDcw (p<0.036) (Fig. A1). The lower repeatability in cascade control could be attributed to the oscillatory nature of muscle force profiles in cascade control (Shah & Kedgley, 2016). However, cascade control resulted in higher muscle forces (p<0.007) as compared to hybrid control (Appendix A.3); therefore, despite the lower repeatability, the coefficient of variation in cascade control was less than 7% for all muscles, for all planar and complex cyclic motions of the wrist, which was similar to that observed in hybrid control (Table A2).


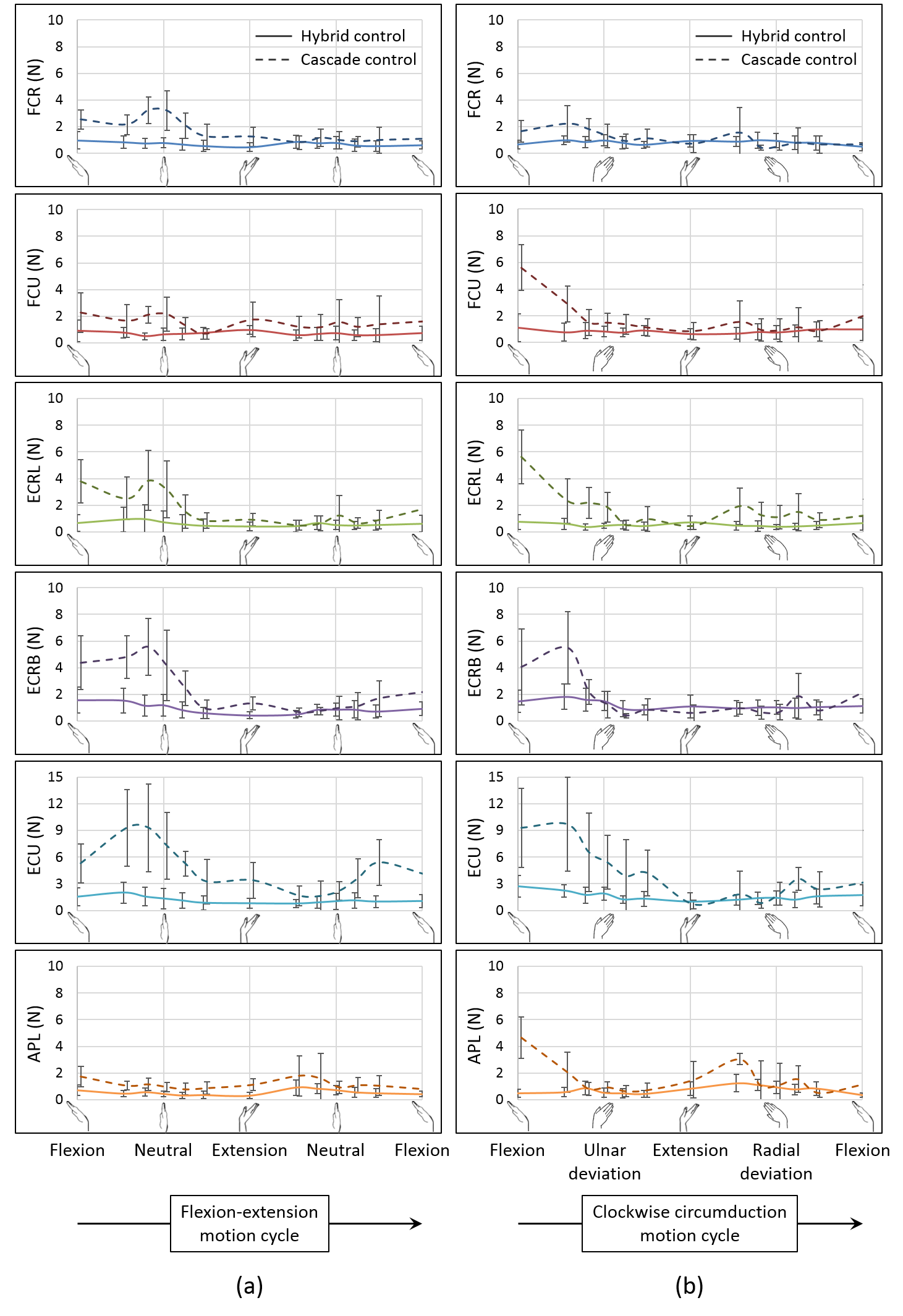


Fig. A1: Repeatability (mean ± standard deviation across 10 specimens) of the muscle forces of the flexor carpi radialis (FCR), flexor carpi ulnaris (FCU), extensor carpi radialis longus (ECRL), extensor carpi radialis brevis (ECRB), extensor carpi ulnaris (ECU) and abductor pollicis longus (APL) for (a) flexion-extension and (b) clockwise circumduction with the hand in the vertically upward orientation in hybrid and cascade control

Table A2: Coefficient of variation of muscle forces, expressed as a percentage of mean force across 10 specimens, in hybrid and cascade control with the hand in the vertically upward orientation. Data is represented as mean ± one standard deviation across the complete range of motion.

| **Motion** | **Control strategy** | **FCR** | **FCU** | **ECRL** | **ECRB** | **ECU** | **APL** |
| --- | --- | --- | --- | --- | --- | --- | --- |
| Flexion-extension (FE) ±30° | Hybrid | 3.9 ± 1.3 | 4.8 ± 1.2 | 3.7 ± 0.6 | 3.1 ± 0.9 | 2.8 ± 0.9 | 3.8 ± 1.1 |
|  | Cascade | 6.6 ± 4.0 | 3.8 ± 1.6 | 3.8 ± 2.2 | 4.0 ± 2.4 | 5.9 ± 2.6 | 4.5 ± 1.4 |
| Radioulnar deviation (RUD) ±10° | Hybrid | 2.4 ± 0.6 | 4.3 ± 1.3 | 2.4 ± 0.5 | 2.9 ± 0.8 | 2.7 ± 0.7 | 3.8 ± 1.6 |
|  | Cascade | 3.8 ± 2.7 | 3.7 ± 1.4 | 3.7 ± 3.7 | 2.6 ± 1.6 | 2.6 ± 1.0 | 6.0 ± 8.1 |
| Dart thrower’s motion  (±20° FE with ±15° RUD) | Hybrid | 3.3 ± 1.5 | 4.2 ± 1.5 | 2.8 ± 0.8 | 2.5 ± 0.6 | 2.4 ± 0.8 | 3.9 ± 1.3 |
|  | Cascade | 3.6 ± 1.9 | 3.9 ± 2.4 | 5.0 ± 5.7 | 2.9 ± 2.5 | 3.1 ± 1.9 | 6.0 ± 8.6 |
| Clockwise circumduction  (±30° FE with ±10° RUD) | Hybrid | 4.8 ± 1.8 | 4.7 ± 0.7 | 3.0 ± 1.0 | 3.5 ± 0.7 | 3.6 ± 1.1 | 4.9 ± 1.5 |
|  | Cascade | 5.0 ± 3.3 | 4.1 ± 3.4 | 3.9 ± 2.8 | 3.0 ± 2.4 | 5.1 ± 3.1 | 6.0 ± 4.6 |
| Anticlockwise circumduction  (±30° FE with ±10° RUD) | Hybrid | 4.5 ± 1.3 | 3.7 ± 1.3 | 4.2 ± 0.7 | 2.1 ± 0.4 | 3.1 ± 0.8 | 4.5 ± 2.0 |
|  | Cascade | 4.5 ± 2.7 | 3.9 ± 3.5 | 2.3 ± 1.3 | 2.2 ± 1.3 | 5.2 ± 2.4 | 5.4 ± 3.7 |

### A.3 – Peak and mean muscle forces

As compared to hybrid control, cascade control resulted in higher peak forces and mean forces for all muscles in all the cyclic motions (p<0.007) (Table A3). This indicated a higher level of co-contraction in cascade control, probably due to differences in the design of the control strategies, particularly the presence of an optimisation routine in cascade control.

The ranges of motion of FE-30 and RUD-10 were selected so that the peak muscle forces from hybrid and cascade control across 10 specimens could be compared to results from other wrist simulators in the literature (Erhart et al., 2012; Werner et al., 1996). In the case of hybrid control, the peak forces of all muscles for both FE-30 and RUD-10 were within two standard deviations of values reported in the literature, except for lower peak force for the FCU and ECRL for FE-30 (Fig. A2). In the case of cascade control, however, the peak muscle forces were higher than two standard deviations when compared to values in the literature, for ECU in FE-30 and all muscles in RUD-10 (Fig. A2). The variations in the muscle force apportioning could be attributed to the different control strategies used to control the wrist joint. While Werner et al. (1996) used position control for agonists and force control for antagonists, and Erhart et al. (2012) used force control for all muscles, the simulator used in this study combined position and force feedback for all muscles to control the joint using hybrid and cascade control (Shah & Kedgley, 2016). There is very little data from in vivo studies, like data from instrumented implants or electromyographic (EMG) studies, in literature to validate the muscle forces obtained from the various physiologic simulators. However, of the two novel control strategies proposed in this study, hybrid control was preferred over cascade control, owing to shorter cycle times (Appendix A.1), higher repeatability (Appendix A.2) and better agreement of muscle forces with values from the literature (Appendix A.3).


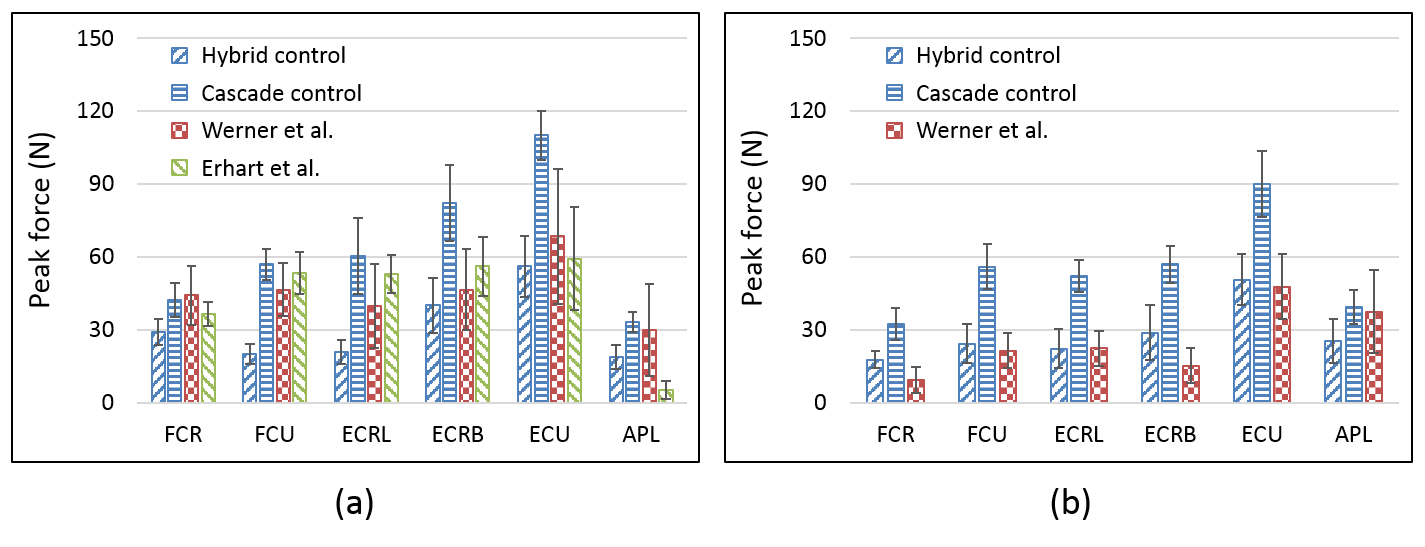


Fig. A2: Comparison of peak muscle forces in hybrid and cascade control with the hand in the vertically upward orientation for (a) flexion-extension of ±30° and (b) radioulnar deviation of ±10° across 10 specimens with data from Werner et al. (1996) for 12 specimens and Erhart et al. (2012) for 6 specimens for the flexor carpi radialis (FCR), flexor carpi ulnaris (FCU), extensor carpi radialis longus (ECRL), extensor carpi radialis brevis (ECRB), extensor carpi ulnaris (ECU), abductor pollicis longus (APL)

Table A3: (a) Peak forces and (b) mean forces during cyclic motions in hybrid control and cascade control with the hand in the vertically upward orientation. Data is represented as mean ± one standard deviation across 10 specimens. (FE-30 = flexion extension of ±30°, RUD-10 = radioulnar deviation of ±10°, DTM = dart thrower’s motion from 20° extension with 15° radial deviation to 20° flexion with 15° ulnar deviation, CCD_cw_ = clockwise circumduction from 30° flexion to 10° ulnar deviation to 30° extension to 10° radial deviation, CCD_acw_ = anticlockwise circumduction from 30° flexion to 10° radial deviation to 30° extension to 10° ulnar deviation)

| (a) | **Motion** | **FCR (N)** | **FCU (N)** | **ECRL (N)** | **ECRB (N)** | **ECU (N)** | **APL (N)** |
| --- | --- | --- | --- | --- | --- | --- | --- |
| Hybrid control | FE-30 | 29.0 ± 5.3 | 20.0 ± 4.3 | 20.8 ± 5.1 | 40.0 ± 11.3 | 56.1 ± 12.6 | 18.8 ± 5.0 |
|  | RUD-10 | 17.6 ± 3.6 | 24.3 ± 8.2 | 22.3 ± 8.2 | 28.8 ± 11.4 | 50.7 ± 10.3 | 25.3 ± 9.0 |
|  | DTM | 24.6 ± 8.9 | 35.6 ± 11.7 | 22.4 ± 7.1 | 39.8 ± 11.6 | 59.5 ± 8.2 | 26.7 ± 14.4 |
|  | CCD_cw_ | 34.3 ± 6.4 | 25.4 ± 11.4 | 26.4 ± 11.6 | 46.1 ± 13.8 | 63.7 ± 12.5 | 27.1 ± 10.9 |
|  | CCD_acw_ | 27.0 ± 6.2 | 35.4 ± 19.1 | 28.5 ± 12.7 | 48.9 ± 15.8 | 65.7 ± 15.8 | 24.0 ± 9.4 |
| Cascade control | FE-30 | 42.1 ± 6.9 | 56.9 ± 6.4 | 60.5 ± 15.7 | 82.2 ± 15.6 | 110 ± 10.0 | 33.1 ± 4.0 |
|  | RUD-10 | 32.4 ± 6.6 | 55.9 ± 9.3 | 52.1 ± 6.5 | 57.0 ± 7.6 | 90.0 ± 13.4 | 39.3 ± 6.9 |
|  | DTM | 38.7 ± 7.6 | 61.7 ± 7.1 | 56.2 ± 6.1 | 61.1 ± 7.2 | 104 ± 12.1 | 40.8 ± 6.5 |
|  | CCD_cw_ | 45.8 ± 7.1 | 55.5 ± 6.3 | 53.0 ± 10.8 | 71.1 ± 14.3 | 110 ± 12.4 | 41.8 ± 4.9 |
|  | CCD_acw_ | 36.3 ± 7.2 | 64.2 ± 5.6 | 63.0 ± 10.7 | 70.8 ± 13.2 | 100 ± 19.8 | 40.6 ± 6.0 |

| (b) | **Motion** | **FCR (N)** | **FCU (N)** | **ECRL (N)** | **ECRB (N)** | **ECU (N)** | **APL (N)** |
| --- | --- | --- | --- | --- | --- | --- | --- |
| Hybrid control | FE-30 | 17.4 ± 2.7 | 13.7 ± 1.9 | 14.5 ± 2.2 | 28.5 ± 7.5 | 40.5 ± 6.4 | 13.1 ± 3.1 |
|  | RUD-10 | 12.9 ± 1.6 | 14.9 ± 2.8 | 15.8 ± 4.0 | 23.3 ± 9.6 | 36.4 ± 6.0 | 15.7 ± 3.3 |
|  | DTM | 16.7 ± 5.1 | 17.1 ± 2.6 | 14.6 ± 2.9 | 29.7 ± 10.6 | 40.4 ± 6.0 | 16.2 ± 5.1 |
|  | CCD_cw_ | 18.3 ± 2.9 | 17.8 ± 8.7 | 17.0 ± 5.4 | 32.1 ± 8.3 | 42.7 ± 6.3 | 14.9 ± 4.8 |
|  | CCD_acw_ | 16.4 ± 3.6 | 21.4 ± 11.3 | 17.2 ± 4.7 | 33.6 ± 10.8 | 41.3 ± 4.4 | 14.5 ± 4.4 |
| Cascade control | FE-30 | 27.1 ± 4.8 | 42.9 ± 6.9 | 42.0 ± 7.0 | 54.7 ± 7.2 | 75.7 ± 8.2 | 24.7 ± 3.7 |
|  | RUD-10 | 26.0 ± 6.1 | 42.9 ± 7.6 | 40.8 ± 6.4 | 50.3 ± 7.3 | 70.2 ± 9.5 | 25.8 ± 4.7 |
|  | DTM | 28.0 ± 6.5 | 45.6 ± 7.2 | 41.0 ± 6.1 | 51.4 ± 6.9 | 73.2 ± 8.1 | 26.7 ± 4.6 |
|  | CCD_cw_ | 26.8 ± 5.5 | 44.3 ± 7.5 | 41.7 ± 6.7 | 53.2 ± 6.8 | 75.2 ± 8.1 | 25.9 ± 4.8 |
|  | CCD_acw_ | 25.6 ± 6.1 | 43.8 ± 7.3 | 42.3 ± 6.3 | 53.4 ± 6.7 | 75.0 ± 8.0 | 25.7 ± 4.6 |
